# Nodes and biological processes identified on the basis of network analysis in the brain of the senescence accelerated mice as an Alzheimer's disease animal model

**DOI:** 10.3389/fnagi.2013.00065

**Published:** 2013-10-29

**Authors:** Xiao-rui Cheng, Xiu-liang Cui, Yue Zheng, Gui-rong Zhang, Peng Li, Huang Huang, Yue-ying Zhao, Xiao-chen Bo, Sheng-qi Wang, Wen-xia Zhou, Yong-xiang Zhang

**Affiliations:** ^1^Department of Neuroimmunopharmacology, Beijing Institute of Pharmacology and ToxicologyBeijing, China; ^2^Department of Biotechnology, Beijing Institute of Radiation MedicineBeijing, China

**Keywords:** Alzheimer's disease, senescence accelerated mouse prone 8, molecular network, hippocampus, cerebral cortex, differential expressed genes, synaptic transmission, apoptosis

## Abstract

Harboring the behavioral and histopathological signatures of Alzheimer's disease (AD), senescence accelerated mouse-prone 8 (SAMP8) mice are currently considered a robust model for studying AD. However, the underlying mechanisms, prioritized pathways and genes in SAMP8 mice linked to AD remain unclear. In this study, we provide a biological interpretation of the molecular underpinnings of SAMP8 mice. Our results were derived from differentially expressed genes in the hippocampus and cerebral cortex of SAMP8 mice compared to age-matched SAMR1 mice at 2, 6, and 12 months of age using cDNA microarray analysis. On the basis of PPI, MetaCore and the co-expression network, we constructed a distinct genetic sub-network in the brains of SAMP8 mice. Next, we determined that the regulation of synaptic transmission and apoptosis were disrupted in the brains of SAMP8 mice. We found abnormal gene expression of RAF1, MAPT, PTGS2, CDKN2A, CAMK2A, NTRK2, AGER, ADRBK1, MCM3AP, and STUB1, which may have initiated the dysfunction of biological processes in the brains of SAMP8 mice. Specifically, we found microRNAs, including miR-20a, miR-17, miR-34a, miR-155, miR-18a, miR-22, miR-26a, miR-101, miR-106b, and miR-125b, that might regulate the expression of nodes in the sub-network. Taken together, these results provide new insights into the biological and genetic mechanisms of SAMP8 mice and add an important dimension to our understanding of the neuro-pathogenesis in SAMP8 mice from a systems perspective.

## Introduction

Alzheimer's disease (AD) is a complex neurodegenerative disease. Despite extensive research, the causal chain of mechanisms underlying AD remains unknown. Currently, there are no effective disease-modifying or preventive therapies, and the only available treatment remains symptomatic to some extent. Studies aimed at identifying the mechanisms associated with AD and new therapeutic treatments are currently being performed in rodent models of AD. Using SAM/resistant-1 (SAMR1) as a control, the senescence accelerated mouse prone-8 (SAMP8) is a robust model of AD because it shares phenotypes that resemble the symptoms of late-onset and age-related sporadic AD patients and because has distinct advantages over the gene-modified model (Pang et al., 2006; Pallas et al., 2008; Woodruff-Pak, 2008; Tomobe and Nomura, 2009; Morley et al., 2012; Pallàs, 2012). Although investigators have developed new therapies and have tested in detail, the structural (Gutierrez-Cuesta et al., 2007; del Valle et al., 2012; Li et al., 2012), functional (Sureda et al., 2006; Tajes et al., 2008; Lou et al., 2012; Yamaguchi et al., 2012) and behavioral consequences (Gong et al., 2008; Shi et al., 2010b; Shih et al., 2010; Chang et al., 2012; Kanno et al., 2012; Lopez-Ramos et al., 2012; Lou et al., 2012; Orejana et al., 2012; Dobarro et al., 2013; Huang et al., 2013; Sawano et al., 2013) of AD-associated pathology based on SAMP8 mice, little progress has been made with regard to the patho-physiological mechanisms of SAMP8 mice, and the underlying causes of the AD-like phenotype in SAMP8 mice remain unknown.

A comprehensive characterization of the biological molecular-network can provide critical insights into the underlying mechanisms. Moreover, identification of the biological molecular pathways may serve as effective targets for therapeutic intervention (Chen et al., 2008; Emilsson et al., 2008; Dobrin et al., 2009; Zhang et al., 2013). Insights into the patho-physiological mechanisms involved in AD are mirrored in the alterations of the molecular network in the brain (Chen et al., 2008; Emilsson et al., 2008; Chan et al., 2012; Liang et al., 2012; Satoh, 2012; Furlong, 2013; Zhang et al., 2013). To understand the molecular underpinnings of SAMP8 as an AD model and to provide insight into the underlying molecular mechanisms of AD, we examined the differentially expressed genes in the hippocampus and cerebral cortex of SAMP8 compared with SAMR1 mice with age. Next, we constructed distinct molecular sub-networks based on the gene expression data and identified numerous functional biological processes and cellular pathways in the brains of SAMP8 mice. Moreover, we highlighted the remarkable modules that were dominated by differentially expressed hub genes in SAMP8 mice. Finally, we identified microRNAs targeting the genes in the distinct sub-network in the brains of SAMP8 mice. Our results present a complex multifactorial basis of the underlying pathophysiology in SAMP8 mice as an AD animal model.

## Materials and methods

**Figure 1:**
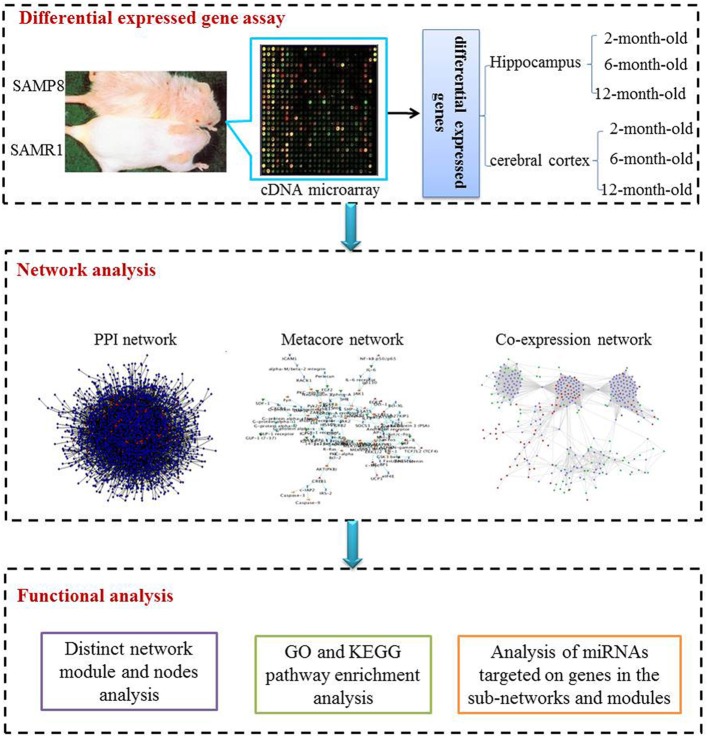
**The scheme of analysis procedure.** The procedure includes three steps. Firstly differential expressed genes in the hippocampus and cerebral cortex of SAMP8 mice comparing with age-matched SAMR1 mice at the age of 2, 6, and 12 months were identified utilizing the cDNA microarray technique. Then, we inferred three sub-networks based on PPI, MetaCore and co-expression network, respectively. Distinct network modules and nodes were identified based on the three sub-networks. For each sub-network and module, GO and KEGG pathway enrichment were achieved employing DAVID. Finally, miRNAs potentially involved in AD-like phenotype in SAMP8 mice were analyzed using experimental validated miRNA target data.

The study included three procedural modules, including differential gene expression assay, network analysis and functional analysis (Figure 1)

### Animals

The original SAMR1 and SAMP8 mice were kindly provided by Dr. T. Takeda at Kyoto University (Kyoto, Japan) and housed at the Beijing Institute of Pharmacology and Toxicology under a natural light-dark cycle (12 h light:12 h dark), room temperature (25 ± 1°C), and normal relative humidity (50 ± 5%). Food and water were provided *ad libitum*. Two-, six- and twelve-month-old male SAMP8 and SAMR1 mice were used. Each group (*n* = 6) was sacrificed by decapitation, and their brains were removed and placed on ice before dissection of the hippocampus and cerebral cortex. Animal care and experimental procedures were performed according to the guide for the Care and Use of Laboratory Animals as adopted by the United States National Institutes of Health.

### Differential gene expression assay using cDNA microarray

A gene expression assay were performed according to previously described methods (Cheng et al., 2007a,b; Zheng et al., 2008). Briefly as follows:

Total RNA was purified from the hippocampus and cerebral cortex of each group using TRIZOL reagent (*Invitrogen* Cat. No. 15596-026). The integrity of the total RNA was detected using agarose gel, and the purity and concentration were determined using the spectrophotometer (NanoDrop, ND-1000).

Fifty micrograms of total RNA were used for reverse transcription according to standard protocols using SUPERSCRIPT III reverse transcriptase (*Invitrogen* Cat. No. 18080-044) and aa-dUTP (Amersham Pharamacia Biotech). Subsequently, aa-cDNA first strand was purified using the QIAquick PCR purification kit (QIAGEN Cat. No. 28104).

The purified aa-dUTP of the control and treated groups were labeled using cy5 or cy3 monofunctional dye (Amersham Pharamacia Biotech), respectively. The corresponding fluorescently labeled cy3 and cy5 cDNA were combined and purified using the MinEluteTM Reaction Cleanup Kit (QIAGEN Cat. No. 28204) and lyophilized in the nucleic dryer (SPD1010 SpeedVac).

The amino groups on the spotted slide (Cheng et al., 2007b) were blocked at room temperature by washing with NaBH_4_/PBS/ethanol. Prehybridization in the hybridization box was performed for 1 h at 42°C, and then plated with buffer containing 5× SSC, 0.1% SDS, 1% BSA under glass coverslips in the hybridization chamber (Robbins Scientific Co.).

After pre-hybridization, the slides were washed and dried immediately. The dried fluorescently labeled cDNA was resolved in 15 μ L hybridization buffer, denatured at 98°C for 5 min and centrifuged at 12,000 rpm for 5 min; the entire supernatant was then pipetted and spread onto the slide. The hybridization procedure was the same as the pre-hybridization procedure except for the 18 h hybridization time. Subsequently, the slides were washed and dried immediately.

The hybridized array was scanned and analyzed using the GenePix 4100Amicroarray scanner (Axon Instruments Inc.) and GenePix Pro 5.1 software (Axon Instruments Inc.), which was combined using Microsoft Excel software. Statistical analyses of each remaining gene on six replicate microarray slides for the experimental repetatur was implemented using SAM software (significance analysis microarray, Stanford University) with one class mode (δ = 0.96938). The absolute value of fold change in gene expression exceeding 1.6 was considered a significant change in expression.

### Network inference procedure

We employed three types of biological networks to infer the most relevant sub-networks in the SAMP8 mice as an AD model, including the background networks; protein-protein interaction (PPI) network, co-expression network and MetaCore network.

#### Protein-protein interaction network analysis

The protein-protein interaction network (PPI) is modeled as an undirected graph with nodes representing the genes, and the edges representing the physical or binding interactions between the proteins encoded by the genes. First, we downloaded the background networks from the Human Protein Reference Database (HPRD, www.hprd.org), containing 9673 proteins and 39,240 interactions (Human Protein Reference Database-2009 update, release 9). Next, to investigate the sub-network in the brains of SAMP8 mice, we employed the algorithm proposed by Alexey et al. (Antonov et al., 2009). In detail, using MATLAB software, we referred to the input gene lists, which were differentially expressed genes in the brains of SAMP8 in the present study, as seed genes, and these seed genes were mapped onto the PPI network. The distance between any two seed genes was calculated. Specifically, this distance indicated the minimal number of consecutive steps from one seed gene to another. For example, a distance of 1 indicated that two seed genes were connected directly, and a distance of 2 indicated that two seed genes were separated by a non-seed gene (known as the intermediate gene). The sub-network in SAMP8 consisted of seed genes whose distance was less than 3, intermediate genes, and their edges (Supplement Figure 1).

#### Metacore network analysis

MetaCore is commercially available software (Thompson Reuters, New York, NY) used for the functional analysis of high throughput data. MetaCore provides the known molecular interactions and pathways curated manually from published papers. We submitted the differentially expressed genes in SAMP8 mice in the present study to MetaCore (https://portal.genego.com, released at August 29, 2012). To obtain a comparatively complete background network for SAMP8 mice, we developed a network employing the differentially expressed genes as seed genes using a different algorithm (shortest path, analysis network, transcript regulation), and integrated these networks according to MATLAB script. The sub-network inference procedure of the MetaCore network in the brains of SAMP8 mice was the same as the PPI network (Supplement Figure 1).

#### Co-expression network analysis

The co-expression network is a phenotypic network based on transcriptional patterns. The human co-expression network was downloaded from COXPRESdb (Obayashi and Kinoshita, 2011) version c4.0, which was released on August 29, 2012 (http://coxpresdb.jp). We selected gene pairs with significant co-expression patterns whose correlation coefficient was equal to or greater than 0.5, and the reserved interactions were used as the background co-expression network. The sub-network inference procedure of the co-expression network in the brains of SAMP8 mice is the same as the PPI network (Supplement Figure 1).

### Analysis of functional categories in the network

#### Distinct network module and nodes analysis

Using MATLAB software, we identified the hub seed genes and key modules in the brains of SAMP8 mice. The seed genes were differentially expressed in SAMP8 mice in the present study. For each of the PPI, co-expression, or MetaCore sub-network, seed genes with more than 14 direct interacting neighbors were defined as hub seed genes. These hub seed genes and their direct interactions formed the key module in each of the PPI, co-expression, or MetaCore sub-network.

#### GO and KEGG pathway enrichment analysis

For the biological and functional annotation of the genes within each PPI, co-expression, or MetaCore sub-network in the brains of SAMP8 mice, Gene Ontology (GO) analysis and Kyoto Encyclopedia of Genes and Genomes (KEGG) pathway enrichment were performed using the Database for Annotation, Visualization and Integrated Discovery (DAVID) (http://david.abcc.ncifcrf.gov/) (Huang da et al., 2009a,b). Using DAVID-based analyses, the reported *P*-values were derived from the Expression Analysis Systematic Explorer (EASE) score probability, which is a modified version of the Fisher's exact test. The significance of the overrepresentation was adjusted for multiple comparisons to control for the false discovery rate (FDR) using the approximated FDR tools provided in DAVID. The GO Biological Processes (BPs), highest-level terms in the BP Gograph structure, and KEGG pathways with *P* < 10^−3^ and FDR <0.01 were considered to be a specific function and biological pathway of genes in the PPI, co-expression, or MetaCore sub-network, and modules in the brains of SAMP8 mice.

#### MicroRNA targeted genes in the sub-network

To identify microRNAs involved in the AD-like phenotype of SAMP8 mice, we obtained 3923 miRNA-target pairs from four validated miRNA target databases updated on April 16, 2013, including Tarbase (http://www.diana.pcbi.upenn.edu/tarbase) (Sethupathy et al., 2006), miR2disease (http://www.miR2Disease.org) (Jiang et al., 2009), miRecords (http://miRecords.umn.edu/miRecords) (Xiao et al., 2009a), and miRtarbase (http://miRTarBase.mbc.nctu.edu.tw) (Hsu et al., 2011). These miRNA-target pairs were used to construct the miRNA-gene bipartite graph network in SAMP8 mice. There are two types of vertices in this bipartite graph network. One vertex represents genes in the PPI, co-expression, and the MetaCore sub-network in the brains of SAMP8, and the other vertex represents the miRNAs targeting these genes.

## Results

### Alterations in gene expression in the hippocampus and cerebral cortex of SAMP8 mice

Differentially expressed mRNA in the hippocampus and cerebral cortex of SAMP8 and SAMR1 mice at 2, 6, and 12 months were investigated using cDNA microarray (Cheng et al., 2007b). We found that the gene expression profile in the hippocampus of SAMP8 mice were different from SAMR1 mice at three different ages (Table 1). In the hippocampus, there were 42 differentially expressed genes in SAMP8 mice compared with SAMR1 mice at the age of two months, 12 genes at six months, and 57 genes at twelve months [parts of the data in 12-month-old mice have been published in 2007 (Cheng et al., 2007b)]. In the cerebral cortex, the gene expression profile in SAMP8 mice was different from SAMR1 mice at only two different ages (Table 2). There were 18 and 32 differentially expressed genes in SAMP8 mice compared with SAMR1 mice at 6 and 12 months of age, respectively, with only one difference present at the age of two months.

**Table 1:** Differentially expressed genes in the hippocampus of SAMP8 and SAMR1 mice with aging^*^.

**2-month-old**				**6-month-old**		**12-month-old**			
**Gene symbol**	**Ratio of P8 *to* R1**	**Gene symbol**	**Ratio of P8 *to* R1**	**Gene symbol**	**Ratio of P8 *to* R1**	**Gene symbol**	**Ratio of P8 *to* R1**	**Gene symbol**	**Ratio of P8 *to* R1**
Amfr	1.98	Sympk	−1.88	Penk1	2.70	Dnchc1	8.51	Rock1	1.71
Fhit	−1.62	Mink1	−1.88	Uqcr	1.94	ARNT2	3.24	Olfr273	1.70
Ercc5	−1.63	Trim3	−1.88	Slc17a7	1.85	Rps21	2.97	Thyn1	1.69
Mast2	−1.66	Dnahc8	−1.93	Ssu72	1.82	Rcn 2	2.81	Vps13c	1.52
Dip3b	−1.68	Dusp12	−1.99	Prr6	1.79	Tspan2	2.57	Ager	−1.59
KDELR1	−1.68	Vps13c	−2.01	Tmem186	1.67	UQCRFS1	2.32	ZNF133	−1.64
Syde1	−1.68	Ticam2	−2.01	MTCO1	1.67	MTCO1	2.28	GANP	−1.66
Ankzf1	−1.71	B3gat1	−2.06	Ankzf1	1.62	Cstn1	2.26	Cdkn2a	−1.68
STUB1	−1.72	NSF	−2.10	DUSP12	1.62	Raf1	2.22	Olfr272	−1.69
Kcns2	−1.74	Acrbp	−2.13	Rps6ka1	−1.60	Kinectin	2.21	cox2	−1.72
Strn4	−1.75	Ihpk1	−2.15	cox2	−1.72	ZNF 238	2.14	Bbox1	−1.73
Thyn1	−1.76	Samm50	−2.15	Ttc3	−2.36	Epha4	2.12	Fyco1	−1.80
Def8	−1.81	Tmem186	−2.19			Kcnh6	2.12	DUSP12	−1.84
Rcn2	−1.81	Olfr272	−2.21			Map3k3	2.12	Ankzf1	−1.85
C1qb	−1.82	Gm11502	−2.23			NF-L	2.11	NSF	−1.87
Prr6	−1.84	Kcnh6	−2.25			ADRBK1	2.09	RAB26	−1.91
Rab26	−1.84	Cpsf3l	−2.25			Eph B6	2.06	Ttc3	−1.91
Sec23b	−1.86	Ttc3	−2.36			Fin14	1.97	Map4k6-pending	−1.96
MTCO1	−1.87	Uqcr	−2.50			Ticam2	1.96	Cacng4	−2.00
Ranbp5	−1.87	Ddx3x	−2.53			Uqcr	1.94	Mapt	−2.01
Snx27	−1.87	Oxr1	−2.58			KDELR1	1.92	Fhit	−2.03
						Ntrk2	1.92	Strn4	−2.07
						Snx27	1.87	Rps19bp1	−2.09
						Tmem186	1.85	NRXN1	−2.17
						Gm11502	1.84	RNase H	−2.74
						Rpl26	1.84	STUB1	−3.14
						Mast2	1.82	Sympk	−3.45
						MTCO3	1.75	CAMK2α	−24.51
						Kpnb3	1.72		

* The fold change of gene expression is greater than 1.6 is considered significant; “+” means up-regulated expression; “−” means down-regulated expression.

**Table 2:** Differentially expressed genes in the cerebral cortex of SAMP8 and SAMR1 with aging^*^.

**6-month-old**		**12-month-old**			
**Gene symbol**	**Ratio of P8 *to* R1**	**Gene symbol**	**Ratio of P8 *to* R1**	**Gene symbol**	**Ratio of P8 *to* R1**
pol	2.61	Ihpk1	−1.64	Dusp12	−1.88
Uqcr	1.97	Vps13c	−1.65	Oxr1	−1.89
Tmem186	1.93	Mast2	−1.66	Nsf	−1.92
Ticam2	1.92	Ngrn	−1.66	Samm50	−1.93
cox2	1.87	Def8	−1.69	Trim3	−1.96
Gm11502	1.73	Phb	−1.71	Amfr	−1.97
C1qb	1.72	C1qb	−1.71	Ttc3	−1.98
Rab26	1.71	Kcns2	−1.71	Kcnh6	−2.03
Clstn1	1.68	Sec23b	−1.73	Uqcr	−2.08
Dusp12	1.68	Acrbp	−1.74	NRXN1	−2.10
Trim3	1.67	Rcn2	−1.75	Gm11502	−2.11
Def8	1.66	Sympk	−1.76	cox2	−2.17
Ihpk1	1.65	Kdelr1	−1.77	Ticam2	−2.19
Ttc3	1.61	Ankzf1	−1.79	Tmem186	−2.29
Vps13c	1.60	Rock1	−1.81		
Slc17a7	−2.13	Prr6	−1.81		
Rn18s	−2.21	Fhit	−1.83		
Rps6ka1	−2.27	Syde1	−1.86		

* The fold change of gene expression is greater than 1.6 is considered significant; “+” means up-regulated expression; “−” means down-regulated expression; there was no difference at the age of two months.

### The distinct genetic sub-network in the brains of SAMP8 mice

#### Sub-network based on the PPI network

**Figure 2:**
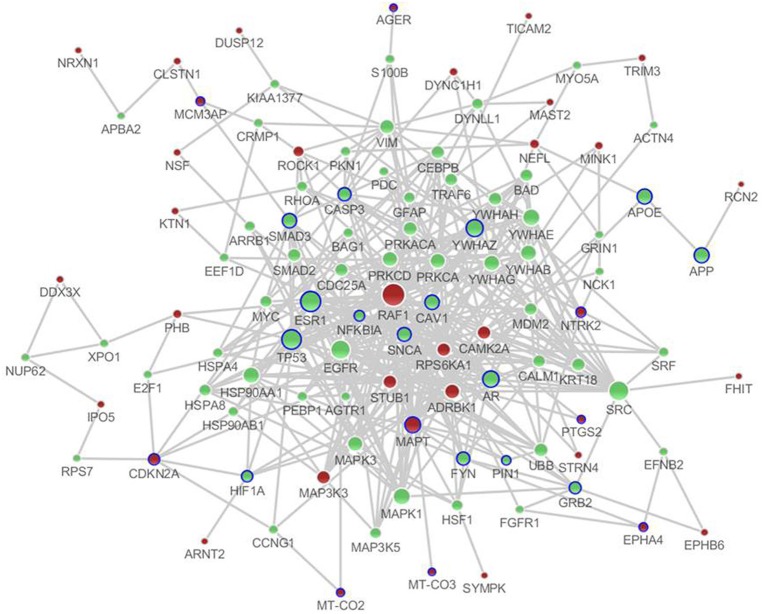
**The distinct genetic sub-network of hippocampus and cerebral cortex in SAMP8 mice based on PPI network.** Red nodes represent differential expressed genes in SAMP8 mice, green nodes are intermediate genes, and genes marked with blue circle are AD-related. The size of the node corresponds to the number of its direct neighbors. The nodes are linked if they have interaction in PPI network.

There were a total of 81 unique differentially expressed genes in the hippocampus and cerebral cortex in SAMP8 mice, including 78 genes in the hippocampus and 37 genes in the cerebral cortex. Using differentially expressed genes in the hippocampus and cerebral cortex, we independently identified the PPI sub-networks of two tissues (Supplement Figure 2A for hippocampus and 2B for cerebral cortex). After constructing the tissue-specific PPI sub-network, we used 81 differentially expressed genes as seed genes to identify the PPI sub-network in SAMP8 mice. These results showed that the sub-network of the hippocampus and cerebral cortex inferred from PPI contained 105 genes, in which 25 genes were AD-related according to AlzGene (http://www.alzgene.org, April 18, 2013) (Bertram et al., 2007) and the *p*-value was 3.744E-14 (fisher exact test) (Figure 2) (Supplement Table 2). Detailed topological information of the sub-networks is provided in the Supplementary materials (Supplement Table 1). Enriched GO terms included the regulation of apoptosis, synaptic transmission, cellular protein complex assembly, protein kinase activity and neuron projection morphogenesis (Figure 5) (Supplement Table 5). However, the neurotrophin signaling pathway, pathways in cancer, MAPK signaling pathway, GnRH signaling pathway, cell cycle, ErbB signaling pathway, oocyte meiosis, adherens junction, long-term potentiation (LTP), AD, focal adhesion, and vascular smooth muscle contraction were enriched (Figure 5) (Supplement Table 5).

#### Sub-network based on the metacore network

**Figure 3:**
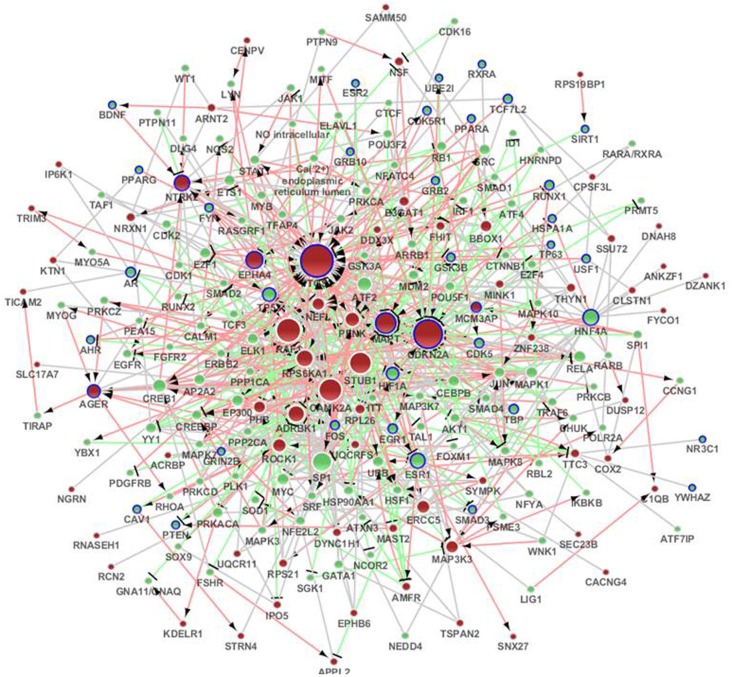
**The distinct genetic sub-network of hippocampus and cerebral cortex in SAMP8 mice based on MetaCore network.** Red nodes represent differential expressed genes in SAMP8 mice, green nodes are intermediate genes, and genes marked with blue board are AD-related. The size of the node corresponds to the number of its direct neighbors. The nodes are linked if they have interaction in MetaCore. The red line with black arrow indicates activation. The blue line with black small line segments indicates inhibition. The blue line indicates there is undefined interaction between nodes.

We submitted the differentially expressed genes in SAMP8 to MetaCore, and the independent sub-networks of hippocampus and cerebral cortex were derived using the MetaCore network as the background network (Supplement Figure 3A for hippocampus and 3B for cerebral cortex). The sub-network for the two tissues contained 213 genes, 40 of which were AD-related (Bertram et al., 2007), and the *p*-value was 2.2 e^16^ (Fisher's exact test) (Figure 3) (Supplement Table 3). Detailed topological information of this sub-network based on MetaCore is provided in the Supplementary materials (Supplement Table 1). Enriched GO terms included the regulation of apoptosis, regulation of synaptic transmission, protein import into nucleus and regulation of the glucose metabolic process (Figure 5) (Supplement Table 5). The KEGG pathways were enriched, including pathways in cancer, the MAPK signaling pathway, neurotrophin signaling pathway, GnRH signaling pathway, Wnt signaling pathway, ErbB signaling pathway, Focal adhesion, TGF-beta signaling pathway, adherens junction, LTP, B cell receptor signaling pathway, chemokine signaling pathway, cell cycle, Toll-like receptor signaling pathway, and the T cell receptor signaling pathway (Figure 5) (Supplement Table 5).

#### Sub-network based on the co-expression network

**Figure 4:**
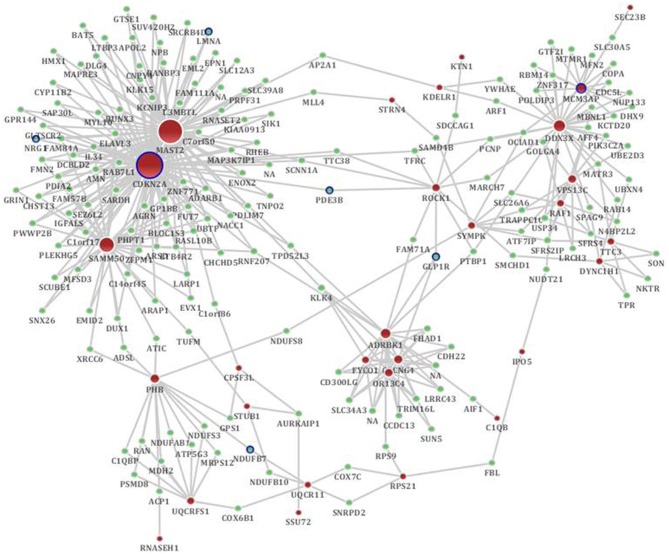
**The distinct genetic sub-network of hippocampus and cerebral cortex in SAMP8 mice based on co-expression network.** Red nodes represent differential expressed genes in SAMP8 mice, green nodes are intermediate genes, and genes marked with blue board are AD-related. The size of the node corresponds to the number of its direct neighbors. The nodes are linked if they are significantly co-expressed.

**Figure 5:**
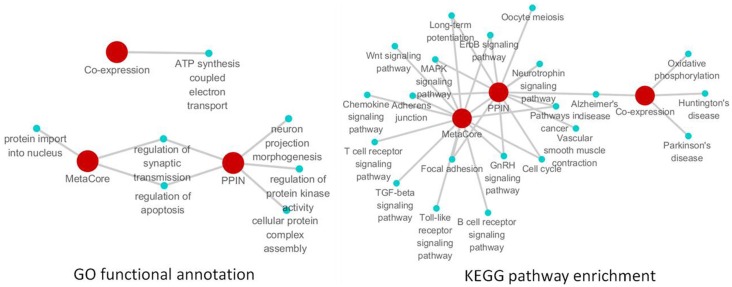
**The remarkable biological processes and pathways in the brain of SAMP8 mice.** GO functional annotation (**left**) and KEGG pathway enrichment (**right**) of PPI, co-expression and MetaCore sub-networks.

According to the sub-network inference procedure described in the PPI network, we derived sub-networks of the hippocampus, cerebral cortex, hippocampus and cerebral cortex using gene pairs with significant co-expression patterns whose correlation coefficient was equal to or greater than 0.5 as the background co-expression network. Independent sub-networks of the hippocampus and cerebral cortex in SAMP8 mice were derived (shown in Supplement Figure 4A for hippocampus and 4B for cerebral cortex). The sub-network for two tissues contained 197 genes, 7 of which were AD-related (Bertram et al., 2007) (Figure 4) (Supplement Table 4). Detailed topological information of the sub-network in SAMP8 mice based on the co-expression network is provided in the Supplementary materials (Supplement Table 1). The ATP synthesis coupled electron transport was enriched using GO terms (Figure 5) (Supplement Table 5). KEGG pathways were enriched, including those involved in Huntington's disease, Parkinson's disease, oxidative phosphorylation and AD (Figure 5) (Supplement Table 5).

#### The remarkable biological processes and pathways in the brains of SAMP8 mice

To investigate the specific function and biological pathway of genes in the PPI, co-expression, and MetaCore sub-network in the brains of SAMP8 mice as an AD animal model, the GO functional annotation and pathway enrichment analysis were performed using the online tool DAVID. Of the biological processes, only the regulation of synaptic transmission and apoptosis was presented in two types of sub-networks in SAMP8 mice at the same time (Figure 5) (Supplement Table 5). This indicated that these two processes are remarkable and might play an important role in the underlying mechanism associated with the AD-like phenotype in SAMP8 mice.

The results of the pathway enrichment analysis showed that in twenty-one cellular pathways, LTP, MAPK signaling pathway, GnRH signaling pathway, ErbB signaling pathway, adherens junction, focal adhesion, cell cycle, pathways in cancer and AD appeared in the PPI and MetaCore or PPI and co-expression sub-network in the brains of SAMP8 mice at the same time (Figure 5) (Supplement Table 5). This indicated that these nine pathways were significant and might contribute to the share cognitive performance and histopathological and biochemical characteristics of AD in SAMP8 mice.

### Significant modules and nodes of the genetic sub-network in the brains of SAMP8 mice

**Figure 6:**
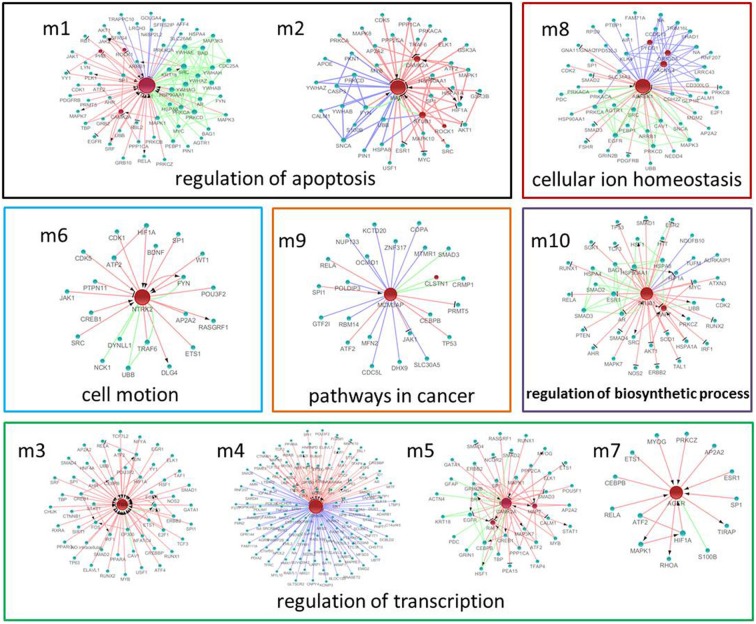
**The significant modules and nodes of genetic sub-network in the brain of SAMP8 mice.** Red nodes are differential expressed genes in SAMP8 mice, and green nodes are intermediate genes. The genes are linked with red line if they have interaction in MetaCore, green line if they have interaction in PPI, and blue line if they are significant co-expressed. The red line with black arrow indicates activation. The red line with black small line segments indicates inhibition.

Using MATLAB software, we identified 10 hub seed genes with more than 14 direct interacting neighbors in the PPI, co-expression, or MetaCore sub-network in the brains of SAMP8, and found 10 modules consisting of hub seed genes and their direct interactions (Figure 6) (Supplement Table 6). These 10 hub seed genes are remarkable nodes in the brains of SAMP8 mice, and include RAF1, MAPT, PTGS2, CDKN2A, CAMK2A, NTRK2, AGER, ADRBK1, MCM3AP and STUB1. The hub nodes of the significant modules m1, m2, m3, m5, m6, and m8 are RAF1, MAPT, PTGS2, CAMK2A, NTRK2, and ADRBK1, respectively, and the hub nodes of m4, m9 and m10 are CDKN2A, MCM3AP, and STUB1, respectively. The hub node of m7 is AGER. This result showed that the abnormal expression of RAF1, MAPT, PTGS2, CDKN2A, CAMK2A, NTRK2, AGER, ADRBK1, MCM3AP, and STUB1 might be involved in the structural, functional, and behavioral consequences of AD-associated pathology in SAMP8 mice.

**Figure 7:**
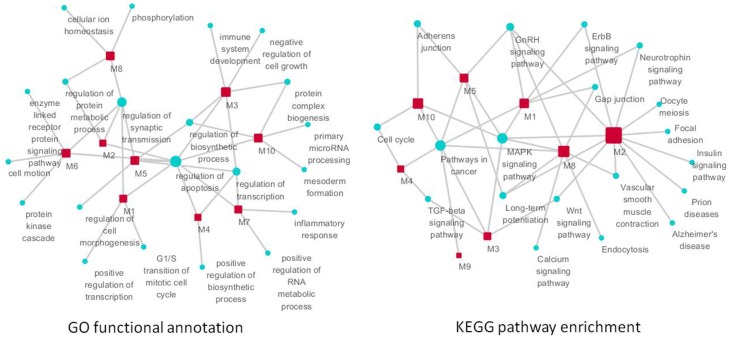
**The function of significant modules and nodes of genetic sub-network in the brain of SAMP8 mice.** GO functional annotation (**left**) and KEGG pathway enrichment (**right**) of 10 modules. Red rectangle nodes are modules, and blue round nodes are GO terms and KEGG pathways, respectively. The size of the node corresponds to its direct neighbors.

GO functional annotation and KEGG pathway enrichment analysis were performed for each module using DAVID. We found that module m1 and m2 were significantly associated with the regulation of apoptosis, and m3, m4, m5 and m7 were associated with the regulation of transcription. In addition, m6, m8, m9, and m10 were associated with cell motion, cellular ion homeostasis, pathways in cancer, and the regulation of biosynthetic process, respectively (Figure 6) (Supplement Tables 6, 7). We highlighted the contribution of the regulation of apoptosis and synaptic transmission in the AD-like phenotype of SAMP8 mice because there were at least five modules that were related to these three biological processes (Figure 7) (Supplement Table 7). This indicated that RAF1, MAPT, PTGS2, CDKN2A, CAMK2A, NTRK2, AGER, ADRBK1, MCM3AP, and STUB1 might induce SAMP8 mice to demonstrate patho-physiological hallmarks of AD by regulating apoptosis and synaptic transmission.

For KEGG pathway enrichment, pathways in cancer, MAPK signaling pathway, GnRH signaling pathway, and LTP are the top four pathways. In addition, the neurotrophin signaling pathway, Wnt pathway, TGF-beta signaling pathway, adherens junction, gap junction, vascular smooth muscle contraction, cell cycle, and ErbB signaling pathway were enriched in two modules (Figure 7) (Supplement Table 7). This finding indicated that RAF1, MAPT, PTGS2, CDKN2A, CAMK2A, NTRK2, AGER, ADRBK1, MCM3AP, and STUB1 might mainly target these 12 pathways, resulting in the typical symptoms of AD observed in SAMP8 mice.

### MicroRNAs targeting the nodes of the genetic sub-network in the brains of SAMP8 mice

**Figure 8:**
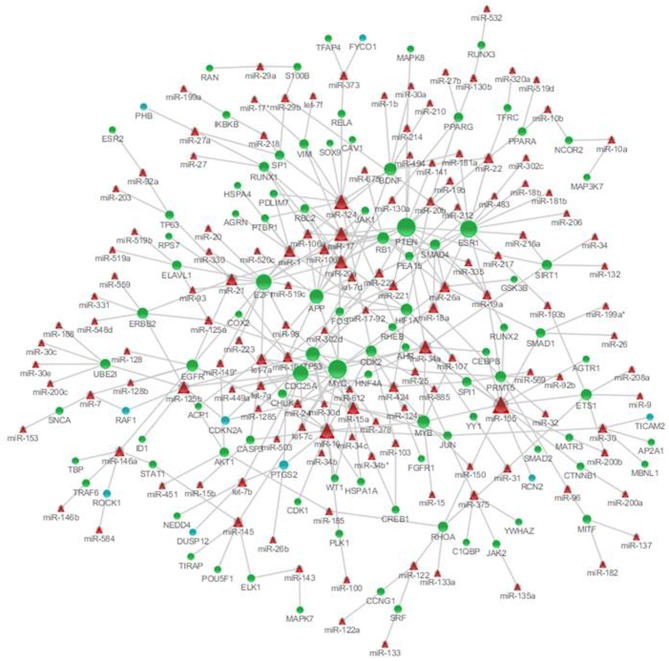
**MicroRNAs targeting on nodes of genetic sub-network in the brain of SAMP8 mice.** In this miRNA-gene bipartite graph network, round nodes represent genes in the PPI, co-expression, and MetaCore sub-network in the brain of SAMP8, blue round nodes are differential expressed genes in SAMP8 mice, and green round nodes are intermediate genes. Red triangle nodes represent miRNAs targeting on these genes. The links represent validated target interactions.

To identify the microRNAs regulating the expression of nodes in the genetic sub-network in the brain of SAMP8 mice, we derived the miRNA-gene bipartite graph network employing four validated miRNA target databases, including the Tarbase, miR2disease, miRecords, and miRtarbase. This miRNA-gene bipartite graph network contained 101 genes, 9 of which were differential expression in SAMP8 mice (Figure 8) (Supplementary Table 8).

The miRNA-gene bipartite graph network in the brains of SAMP8 mice contained 137 miRNAs. These microRNAs regulated the expression of nodes in the genetic sub-network in the brains of SAMP8 mice. The top 10 miRNAs with *P* ≤ 8.37 e^5^ were listed in Table 3. They are miR-20a, miR-17, miR-34a, miR-155, miR-18a, miR-22, miR-26a, miR-101, miR-106b, and miR-125b, indicating that these ten miRNAs could regulate the expression of nodes (genes) in the sub-network of SAMP8 mice and might be one cause inducing SAMP8 mice to exhibit significant nodes (or genes) and to display a distinct genetic sub-network in the brain.

**Table 3:** **The top ten microRNAs targeting on nodes of genetic sub-network in the brain of SAMP8 mice**.

**miRNA**	**Number of targets in miRNA-gene bigraph network**	***P*-value**
miR-20a	9	8.16E-09
miR-17	10	1.30E-07
miR-34a	9	2.78E-07
miR-155	14	2.16E-07
miR-18a	5	4.04E-06
miR-22	5	6.18E-06
miR-26a	6	9.29E-06
miR-101	5	3.30E-05
miR-106b	5	3.30E-05
miR-125b	8	8.37E-05

## Discussion

It is well known that AD is a complex disease and devastating neurodegenerative disorder without effective disease-modifying or preventive therapies. Its progression in late-onset AD studies is fundamentally limited by our reliance on mouse models of severe familial/early-onset AD (Zhang et al., 2013). SAMP8 mice, a spontaneous animal model of accelerated aging and harboring the behavioral and histopathological signatures of AD, may more closely represent the complexity of the disease compared to the gene-modified model due to its multifactorial nature of late-onset or age-related sporadic AD (Pang et al., 2006; Pallas et al., 2008; Woodruff-Pak, 2008; Tomobe and Nomura, 2009; Morley et al., 2012; Pallàs, 2012). Thus, understanding the underpinnings of the AD-like phenotype in SAMP8 mice is essential for developing and evaluating therapeutic approaches for this widespread and devastating insidious disease. A comprehensive characterization of gene-network connectivity and its regulation and association to disease can provide critical insights into the underlying mechanisms, highlight and prioritize pathways, and identify genes and microRNAs that may serve as effective targets for therapeutic intervention (Furlong, 2013; Gandy and Heppner, 2013; Jahanshad et al., 2013; Rhinn et al., 2013; Zhang et al., 2013). The present study identified the sub-network, bioprocesses, pathways, genes and miRNAs in SAMP8 mice employing approaches in network biology, and offered valuable insights for the patho-physiological molecular mechanisms and drug targets or treatments of AD.

### The distinct sub-network in the brains of SAMP8 mice

Although a number of alterations in gene expression and protein abnormalities studies have been performed on SAMP8 mice (Butterfield and Poon, 2005; Carter et al., 2005), no clear picture has emerged on the underlying mechanisms, prioritized pathways and genes in SAMP8 mice linked to AD. With the development of the molecular network analysis method, there have been few progress obtained. For example, using proteomics and network analyses, 17 neuronal and 14 astrocytic differentially expressed proteins in SAMP8 mice were found, and network analysis suggested that the neuronal changes were more complex and had a greater influence (Diez-Vives et al., 2009). In the present study, using three different network analysis methods, we constructed a genetic sub-network using differentially expressed genes in the hippocampus and cerebral cortex of SAMP8 compared with SAMR1 at the age of 2, 6, and 12 months. Based on this sub-network, the underlying mechanisms, prioritized pathways and genes have been identified in SAMP8 mice as an AD model.

### Remarkable biological processes in the brains of SAMP8 mice

In the present study, based on sub-network analysis, biological processes including the regulation of synaptic transmission and apoptosis were identified as disrupted in the brains of SAMP8 mice (Figure 5). This finding suggested that the aberrations in the synaptic transmission and apoptosis may be the underlying mechanism of SAMP8 mice as an AD animal model.

Our previous study demonstrated that the glutamatergic synaptic transmission was weakened in the brains of SAMP8 mice owing to the low protein expression level of vesicular glutamate transporter1 (VGLUT1), a specific marker for glutamatergic neurons, and synaptophysin (Syp), a marker of the synapse (Cheng et al., 2011). Moreover, there were aberrations in morphological synaptic plasticity, including a reduction in the number of apical dendritic thorns, abnormal ultrastructure of excitatory synapses, and a decrease in the expression of GluN1 subunit-containing N-methyl-d-aspartate receptors (NMDARs) and Syn in the hippocampus of SAMP8 mice (Li et al., 2013). In addition, the protein levels of the three representative proteins of synaptic function and synaptic structure, including brain-derived neurotrophic factor (BDNF), post-synaptic density protein-95 (PSD95) and Ca^2+^/calmodulin-dependent protein kinase II (CaMKII) in SAMP8 mice were lower compared to the SAMR1 mice (Li et al., 2009). These results indicated that the aberrations in synaptic transmission emerged in the brains of SAMP8 mice.

For the regulation of apoptosis, there were significant levels of neuronal apoptosis were observed in the hippocampus of SAMP8 mice, which was comparable with those in the SMAR1 mice (Ma et al., 2011; Lou et al., 2012). The number of hippocampal neurons was decreased in the hippocampus of SAMP8 mice (Yaguchi et al., 2010; Hu et al., 2012), and there was an increase in the number of condensed nuclei in primary culture cerebellar neurons from SAMP8 mice (Tajes et al., 2008). Furthermore, there was an age-related significant increase in apoptosis in the medial neocerebellum and vermis, and some Purkinje cells appeared to disappear during aging in SAMP8 mice (Zhu et al., 2007).

### The extraordinary cellular pathways in the brains of SAMP8 mice

The present study clearly showed that the SAMP8 mice involved a dysfunction of many cellular pathways, including LTP, focal adhesion, MAPK signaling pathway (Xiao et al., 2009b), GnRH signaling pathway (Yuan et al., 2005), ErbB signaling pathway, adherens junction (Ueno et al., 2001), cell cycle (Casadesus et al., 2012) and pathways in cancer (Gutierrez-Cuesta et al., 2008) (Figure 5). Genes in the distinct sub-network of the SAMP8 mice might mainly target on these cellar pathways to induce the malfunction of these biological process. Six pathways have been previously reported except for the ErbB signaling pathway and focal adhesion.

For LTP, our previous study showed that the LTP in the CA1 area of hippocampal slices prepared from 2-, 6-, and 12-month-old SAMP8 mice had significantly decreased with age. In addition, LTP in the slices of 6- and 12-month-old mice was markedly decreased below that of the age-matched normal SAMR1 mouse strain (Yang et al., 2005; Hu et al., 2012; Huang et al., 2012). Lopez-Ramos et al. demonstrated that SAMP8 mice presented a significant deficit in LTP at the CA1-medial prefrontal synapse at 5 months of age (Lopez-Ramos et al., 2012). LTP, a well-known form of synaptic plasticity, is the neuronal mechanism underlying learning and memory processes. Consistent with SAMP8 mice, alterations in LTP contribute to the deterioration of learning and memory in single-transgenic (APP751SL), single-transgenic (PS1M146L), double-transgenic (APP751SL/PS1M146L) (Gruart et al., 2008) and triple-transgenic (PS1/M146V, APPSwe, and tauP301L) mice (Garcia-Mesa et al., 2011).

These results showed that, by using the network analysis method, the known cellular pathways were deeply elucidated and the abnormal unknown biological pathways were predicted in SAMP8 mice based on the sub-network, such as the ErbB signaling pathway and focal adhesion.

### Significant genes in the brains of SAMP8 mice

AD is a multifactorial complex disease, and both genetic variants and environmental factors contribute to this disease. The genes more frequently related to AD include APP (Levy et al., 1990; Goate et al., 1991; Goate, 2006), PS-1 (Sherrington et al., 1995; Pantieri et al., 2005; Edwards-Lee et al., 2006; Müller et al., 2013), PS-2 (Levy-Lahad et al., 1995a,b; Rogaev et al., 1995), TREM2 (Guerreiro et al., 2013; Jonsson et al., 2013), CLU, CR1 and PICALM (Harold et al., 2009; Carrasquillo et al., 2010; Corneveaux et al., 2010; Jun et al., 2010; Barral et al., 2012), ABCA7, MS4A4A/MS4A6E, EPHA1, CD33 and CD2AP (Hollingworth et al., 2011; Naj et al., 2011), BIN1 (Lambert et al., 2011; Lee et al., 2011; Wijsman et al., 2011), IL-12/IL-23 (Vom Berg et al., 2012) and APOE (Bickeboller et al., 1997; Elias-Sonnenschein et al., 2011; Genin et al., 2011; Verghese et al., 2011).

Many studies on the differentially expressed genes have been performed in SAMP8 mice, and these data are summarized by Butterfield and Poon (Butterfield and Poon, 2005) and Tomobe and Nomura (Tomobe and Nomura, 2009). Similar to senile plaques, Aβ deposits are one of the pathological hallmarks of AD and Aβ generated from APP in the amyloidogenic pathway. The nucleotide sequence of APP in SAMP8 mice does not have mutations similar to those that have been reported in human familial AD (Kumar et al., 2001). The SAMP8 PS1 cDNA sequence is identical to that of normal mice (Kumar et al., 2009). Using the RNase protection assay, Morley et al. found an increase in APP mRNA in the hippocampus of SAMP8 mice (Morley et al., 2000). Using the RT-PCR technique, Wei et al. demonstrated that the levels of PS-1 mRNA were normally expressed, while PS-2 was significantly higher in the hippocampus of SAMP8 mice compared with age-matched SAMR1 mice (Wei et al., 1999). In the present study, we did not observe a change in the expression of App, PS-1 and PS-2 in the brains of SAMP8 mice using cDNA microarray. Our results on APP and PS-2 mRNA expression were not consistent with those obtained by Wei et al. (1999) and Morley et al. (2000), respectively, which may be due to the methodological or short half-life of the two genes in SAMP8 mice. Additional, Lucas et al. identified the lack of DNA polymerase μ in increased learning and brain LTP in aged mice, where brain aging was delayed in Pol μ^−/−^ mice (Lucas et al., 2013). However, we did not observe an increase in DNA polymerase μ mRNA in SAMP8 mice. This may be due to SAMP8 mice not being a genetic manipulation model. In this study, we observed 81 unique differentially expressed genes in the hippocampus and cerebral cortex of SAMP8 mice, including 78 genes in the hippocampus and 37 genes in the cerebral cortex.

The hub nodes (nodes with a very large number of direct interacting neighbors) are thought to play an important role in biological networks (Jeong et al., 2001; Tew et al., 2007). The present study identified 10 hub genes in the brain of SAMP8 mice, including RAF1 (Wang et al., 2003; Cheng et al., 2007b; Ponomarev et al., 2007; Tseveleki et al., 2010), MAPT (Canudas et al., 2005), PTGS2 (Hoozemans and O'Banion, 2005; Zhang et al., 2005; Ma et al., 2008; Shi et al., 2010a), CDKN2A, CAMK2A, NTRK2, AGER, ADRBK1 (Obrenovich et al., 2006; Cheng et al., 2007b; Degos et al., 2013), MCM3AP and STUB1 (Cheng et al., 2007b; Zhang et al., 2008) (Figure 6). These ten hub genes in SAMP8 mice were different from the genes more frequently related to the AD patient. This may be due to SAMP8 mice being a spontaneous animal model and not a gene-modified model of AD. Moreover, the phenotypes of SAMP8 mice resembled the symptoms of the late-onset and age-related sporadic AD patient (Pallas et al., 2008). In these genes, we first identified the gene expression of CDKN2A and MCM3AP, which were changed in SAMP8 mice.

The present study showed cyclin-dependent kinase inhibitor 2A (CDKN2A) was down-regulated in the hippocampus of SAMP8 mice. Zuchner and colleagues identified that CDKN2A at 9p21 are implicated in the susceptibility in the late-onset AD (Zuchner et al., 2008), but the role of CDKN2A genetic variants in AD is not confirmed in late-onset patients based on Tedde and colleagues's (2011). Therefore, further studies are needed to elucidate the role of tumor suppressor protein CDKN2A in the susceptibility of AD.

The protein encoded by the gene minichromosome maintenance complex component 3 associated protein (MCM3AP, also known as GANP) is a minichromosome maintenance protein 3 (MCM3) binding protein. MCM3 is one of the MCM proteins essential for the initiation of DNA replication. MCM3 binding protein was demonstrated to be an acetyltransferase that acetylates MCM3 and is an inhibitor of DNA replication initiation (Takei et al., 2002, 2001). The present study showed MCM3AP was down-regulated in the hippocampus of SAMP8 mice.

Calcium/calmodulin-dependent protein kinase II-alpha (CAMK2A) is one of the most abundant subunits of the calcium/calmodulin-dependent protein kinase II in the cerebral cortex and hippocampus, is required for hippocampal LTP and is closely linked to AD. Our previous study showed that the levels of CAMK2A mRNA and protein were abnormal in the hippocampus and cerebral cortex of SAMP8 mice (Cheng et al., 2007b; Zhang et al., 2009). In addition, other data showed that the CAMK2A-containing neurons were selectively lost in the CA1 subfield of AD hippocampus and was accompanied with enhanced CAMK2A in the remaining neurons. Approximately 33% hyperphosphorylated tau-containing neurons were also immunoreactive for CAMK2A. Moreover, CAMK2A was largely deposited in the senile plaques of the AD hippocampus (Wang et al., 2005).

The NTRK2 gene encodes tropomyosin-related kinase B (TrkB), a member of the neurotrophic tyrosine receptor kinase (NTRK) family. TrkB is a membrane-bound receptor that, upon neurotrophin binding, phosphorylates itself and members of the MAPK pathway. BDNF-mediate activation of TrkB initiates three major signaling pathway cascades: phospholipase C, phosphatidylinositol 3-kinase (PI3K), and extracellular signal-regulated kinase (ERK). Our previous data showed that the mRNA expression of NTRK2 was up-regulated in the hippocampus of SAMP8 mice (Cheng et al., 2007b). TrkB decreased in the hippocampus and cerebral cortex early in the progression of AD (Schindowski et al., 2008; Zuccato and Cattaneo, 2009), and has a role in the pathogenesis of AD (Minichiello, 2009; Nagahara et al., 2009; Devi and Ohno, 2012). Currently, the dynamics of BDNF-TrkB signaling and its effect on the downstream signaling events during different forms of LTP are not very well understood. However, an LTP-like increase in the hippocampal synaptic responses was observed in behavioral mice (Gruart et al., 2006), and blocking protein synthesis and BDNF expression in the hippocampus caused a deficit in the persistence of long-term memory (LTM) storage, but not in memory formation (Bekinschtein et al., 2007). Moreover, the TrkB–PLCγ site-activated molecular pathway underlies both associative learning and LTP, which was triggered at the CA3–CA1 hippocampal synapse in behavioral mice (Minichiello et al., 2002; Gruart et al., 2007).

The advanced glycosylation end product-specific receptor (AGER) is a multiligand membrane receptor, and one of its ligands is Aβ (Yan et al., 1996). AGER is the main factor mediating Aβ cytotoxicity (Wan et al., 2013). AGER is a representative influx transporter of APP or Aβ in cerebral vessels, while low-density lipoprotein receptor (LDLR) and LDL-related protein 1 (LRP1) are efflux transporters. The present study showed that AGER was down-regulated in the hippocampus of SAMP8 mice. In addition, other studies have shown that the gene and protein expressions of RAGE were lower in SAMP8 brains compared to SAMR1 mice, while LDLR was higher in SAMP8 brains compared to SAMR1 mice (Wu et al., 2009). In addition, it has been shown that sRAGE is present at lower levels in the blood and brain of AD patients (Emanuele et al., 2005; Nozaki et al., 2007).

### Important microRNAs in the brain of SAMP8 mice

Based on the miRNA-gene bipartite graph network in the brain of SAMP8 mice, we identified the top 10 miRNAs with *P* ≥ 8.37E-05, including miR-20a, miR-17, miR-34a, miR-155, miR-18a, miR-22, miR-26a, miR-101, miR-106b, and miR-125b (Table 3). Many studies have indicated that these miRNAs played an important role in AD by regulating the expression of specific genes. In SAMP8 mice, these ten miRNAs might regulate the abnormal expression of genes (nodes) in the genetic sub-network in the brains of SAMP8 mice, and thereby results in the abnormalities observed in the cellular function and malfunction of the biological process. In these miRNAs, we first indicated that miR-34a, miR-155, miR-18a, miR-22, miR-26a, miR-101, miR-106b, and miR-125b were important in SAMP8 mice.

APP expression was regulated by miR-20a (Hebert et al., 2009; Fan et al., 2010; Delay et al., 2011), miR-17 (miR-17-5p) (Delay et al., 2011), miR-106b (Hebert et al., 2009), and miR-101 (Vilardo et al., 2010; Long and Lahiri, 2011). In addition, a tight correlation between miR-20a/miR-106b and APP was found during brain development and in differentiating neurons (Hebert et al., 2009). Moreover, the miR-17 and miR-20a bindings sites located in or near the APP 3'UTR variants T117C, A454G and A833C, and the A454G variant increased miR-20a binding (Delay et al., 2011), and miR-17 and miR-20a were down-regulated in age-related and senescence-related cellular processes (Hackl et al., 2010).

MiR-34a has been shown to be a direct target of p53 (Chang et al., 2007; He et al., 2007; Raver-Shapira et al., 2007) and YY1, a negative regulator of p53 (Chen et al., 2011). In mice, miR-34a is ubiquitous with the highest expression in the brain, and overexpression of miR-34a in neuroblastoma cell lines modulates neuronal-specific genes (Wei et al., 2008; Chen et al., 2011). MiR-34a regulates the expression of a number of synaptic proteins, in particular, synaptotagmin I and syntaxin 1A in cortical neurons (Agostini et al., 2011). Moreover, miR-34a was significantly up-regulated in sporadic AD subjects (Schipper et al., 2007; Cogswell et al., 2008) and animal models (Li et al., 2011).

MiR-155 is one of the most studied miRNAs and the first miRNA to be described as oncogenic (Costinean et al., 2006). MiR-155 was shown to participate in the regulation of immunological responses and apoptotic pathways (Park and Peter, 2008). MiR-155 is thought to be essential in the pathogenesis of AD according to induced down-regulation of complement factor-H (CFH), an important repressor of the innate immune response (Lukiw et al., 2012).

MiR-18a targets the protein inhibitor of activated STAT-3 (PIAS-3), an inhibitor of STAT-3 signaling (Brock et al., 2011), and TNF-α induced protein 3 (TNFAIP-3), as the NF-κ B pathway inhibitor (Trenkmann et al., 2013), and ataxia telangiectasia mutated (ATM) kinase, as the primary sensor and transducer of DNA damage signal (Song et al., 2011). MiR-18a is involved in the up-regulation of both the constitutive and TNF-α induced secretion of MMP-1 and inflammatory cytokines and chemokines (Trenkmann et al., 2013).

MiR-22 targets the pro-apoptotic activities of mitogen-activated protein kinase 14/p38 (MAPK14/p38) and tumor protein p53-inducible nuclear protein 1 (Tp53inp1). Overexpression of miR-22 decreased neurodegeneration in an *in vitro* model (Jovicic et al., 2013). In addition, miR 22 regulates the cell cycle length in cerebellar granular neuron precursors (Berenguer et al., 2013).

MiR 26a targets phosphatase and tensin homolog (PTEN) (Liu et al., 2012; Li and Sun, 2013). The number and distribution of neurites was markedly increased by miR-26a, and inhibition of miR-26a functioned in attenuated neuronal outgrowth (Li and Sun, 2013).

MiR-125b is involved in the targeting and down-regulation of complement factor-H (CFH) mRNA and CFH expression. MiR-125b was significantly found to be up-regulated in AD brain tissues (Lukiw, 2007; Lukiw and Pogue, 2007; Lukiw and Alexandrov, 2012), and it was proposed to be involved in driven pathogenic signaling in neurodegenerative diseases, including human prion disease and Down's syndrome (Wang et al., 2010; Lukiw and Alexandrov, 2012).

In addition, it was reported that miR-101 regulates ataxin1 expression in the hippocampus (Lee et al., 2008). MiR-106b significantly decreased the ATP-binding cassette transporter A1 (ABCA1) levels and impaired cellular cholesterol efflux in neuronal cells (Kim et al., 2012), targets TGF-β type II receptor (Tβ R II) to affect TGF-β signaling, thereby contributing to the pathogenesis of AD (Wang et al., 2010).

Taken together, aberrations in the normal cellular function and malfunction of biological process were caused by hub genes and their regulated miRNAs disrupt the fine-tuning of genetic networks and caused the AD-like phenotype in SAMP8 mice.

## Conclusions

In summary, in this study, we provided a systems biological interpretation of the molecular underpinnings of SAMP8 mice as an AD animal model based on network analysis. Biological processes, including the regulation of synaptic transmission and apoptosis, were clearly identified as disrupted in the brains of SAMP8 mice. The abnormal expression of gene RAF1, MAPT, PTGS2, CDKN2A, CAMK2A, NTRK2, AGER, ADRBK1, MCM3AP, and STUB1 might be the key cause to the malfunction of biological processes in the brains of SAMP8 mice. In addition, microRNAs, including miR-20a, miR-17, miR-34a, miR-155, miR-18a, miR-22, miR-26a, miR-101, miR-106b, and miR-125b might regulate the expression of genes (nodes) in the sub-network, thereby disrupting the fine-tuning of genetic networks in SAMP8 mice. These results provide new insights into the biological and genetic mechanisms of SAMP8 mice, and add an important dimension to our understanding of the neuropathogenesis in SAMP8 mice from a systems perspective. Moreover, in the extraordinary cellular pathways of the gene involved, we first indicated that the ErbB signaling pathway and focal adhesion in SAMP8 mice were abnormal. Furthermore, the gene expression of CDKN2A and MCM3AP were changed, and miRNAs, including miR-34a, miR-155, miR-18a, miR-22, miR-26a, miR-101, miR-106b, and miR-125b are important in SAMP8 mice in the present study.

## Author contributions

This study was designed by Wen-xia Zhou, Yong-xiang Zhang, and Xiao-rui Cheng. Differentially expressed genes were detected by Xiao-rui Cheng, Yue Zheng, and Huang Huang, and confirmed by Xiao-rui Cheng, Gui-rong Zhang and Yue-ying Zhao. Network analysis was performed by Xiu-liang Cui, Peng Li, Xiao-chen Bo and Sheng-qi Wang and interpreted by Xiu-liang Cui and Xiao-rui Cheng. This paper was written by Xiao-rui Cheng, Xiu-liang Cui, and Wen-xia Zhou. All authors read and approved the final version of the manuscript.

### Conflict of interest statement

The authors declare that the research was conducted in the absence of any commercial or financial relationships that could be construed as a potential conflict of interest.
