# Supplementary figures and images for: Nodes and biological processes identified on the basis of network analysis in the brain of the senescence accelerated mice as an Alzheimer's disease animal model
Source: Front Aging Neurosci. 2013 Oct 29;5:65. doi: 10.3389/fnagi.2013.00065 (PMC3810591; doi:10.3389/fnagi.2013.00065)

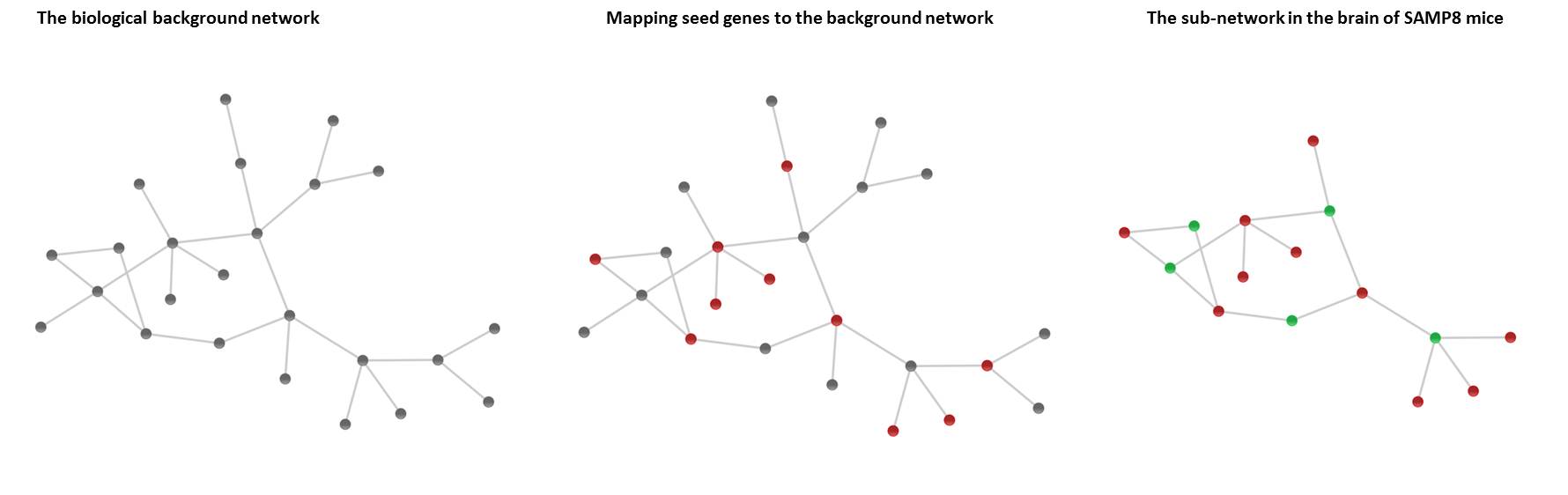

Supplement: Supplementary file 1 [file DataSheet1.ZIP › Supplementary information and Figure legends/supplement figure 1.JPG]

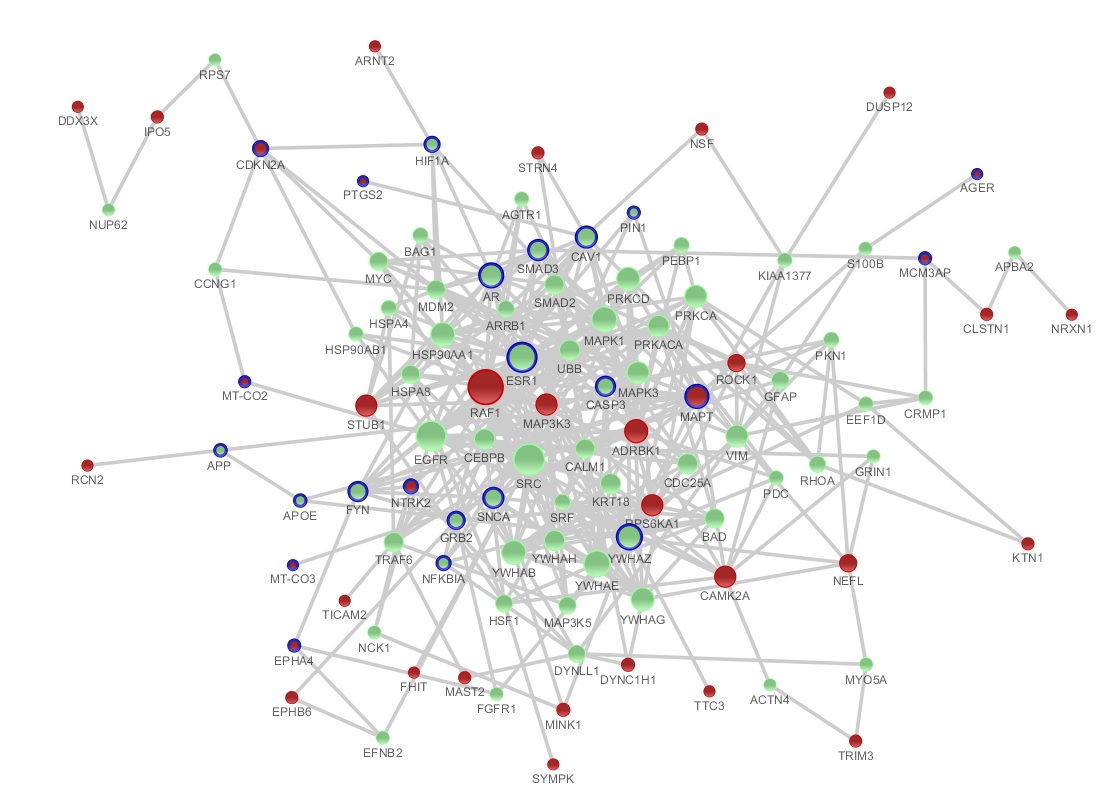

Supplement: Supplementary file 1 [file DataSheet1.ZIP › Supplementary information and Figure legends/supplement figure 2A.JPG]

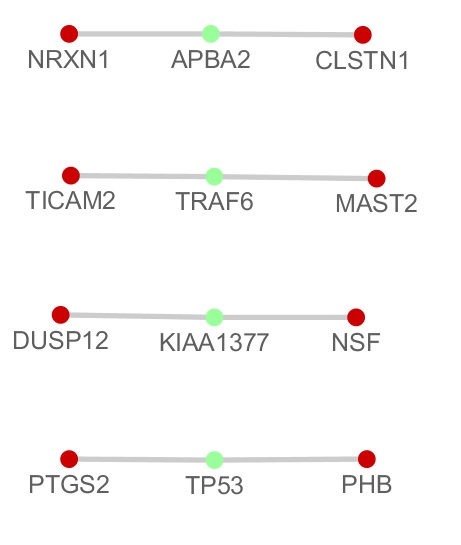

Supplement: Supplementary file 1 [file DataSheet1.ZIP › Supplementary information and Figure legends/supplement figure 2B.JPG]

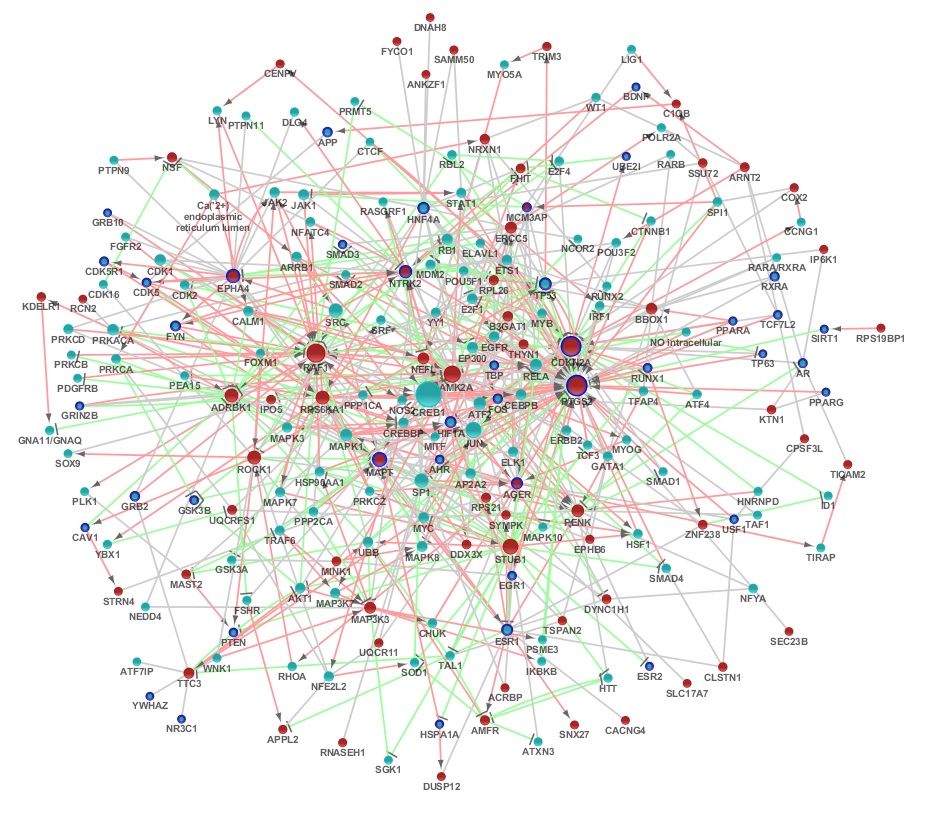

Supplement: Supplementary file 1 [file DataSheet1.ZIP › Supplementary information and Figure legends/supplement figure 3A.JPG]

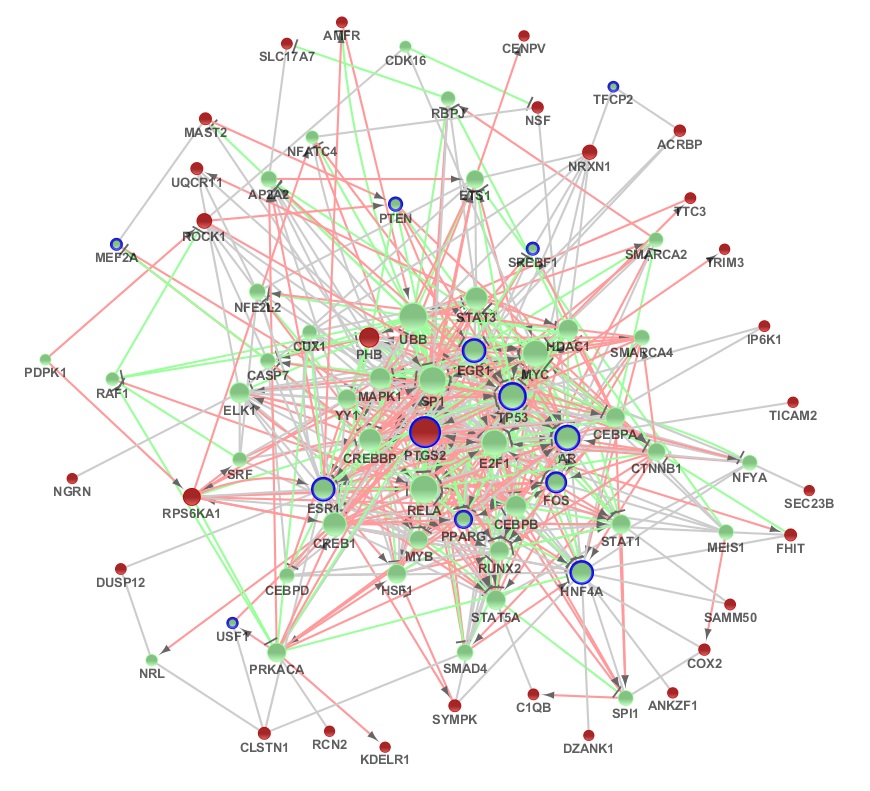

Supplement: Supplementary file 1 [file DataSheet1.ZIP › Supplementary information and Figure legends/supplement figure 3B.JPG]

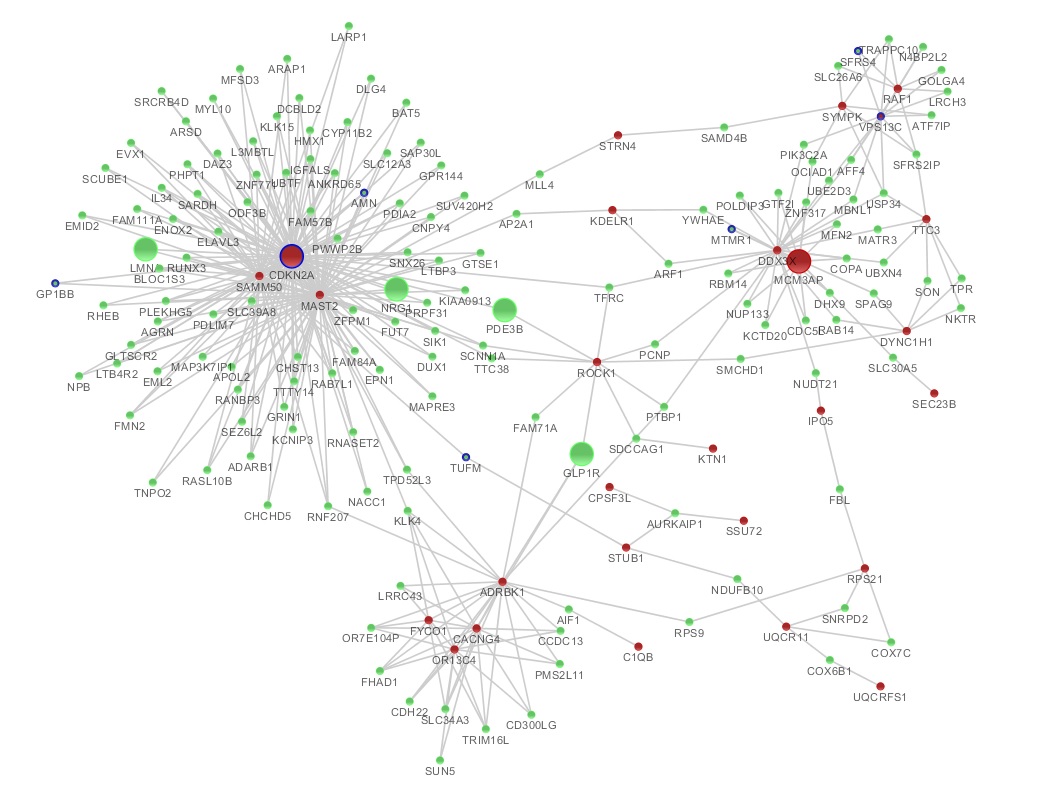

Supplement: Supplementary file 1 [file DataSheet1.ZIP › Supplementary information and Figure legends/supplement figure 4A.JPG]

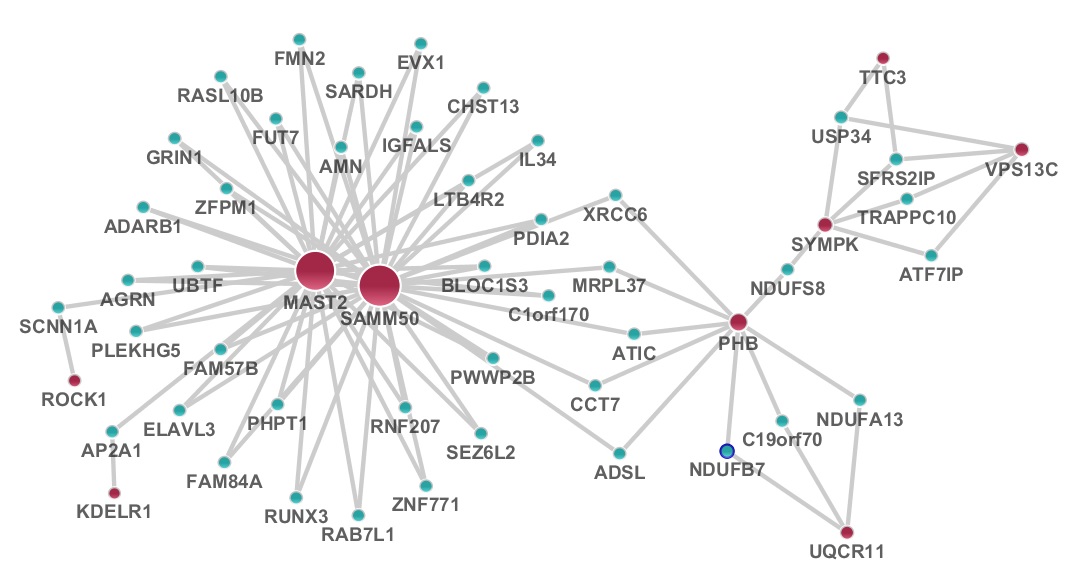

Supplement: Supplementary file 1 [file DataSheet1.ZIP › Supplementary information and Figure legends/supplement figure 4B.JPG]
