# Supplementary material for: Nodes and biological processes identified on the basis of network analysis in the brain of the senescence accelerated mice as an Alzheimer's disease animal model
Source: Front Aging Neurosci. 2013 Oct 29;5:65. doi: 10.3389/fnagi.2013.00065 (PMC3810591; doi:10.3389/fnagi.2013.00065)
Supplement: Supplementary file 1 [file DataSheet1.ZIP › Supplementary information and Figure legends/Figure legends.doc]

**Figure legends**

**Figure 1. The scheme of analysis procedure.** The procedure includes three steps. Firstly differential expressed genes in the hippocampus and cerebral cortex of SAMP8 mice comparing with age-matched SAMR1 mice at the age of 2, 6, and 12 months were identified utilizing the cDNA microarray technique. Then, we inferred three sub-networks based on PPI, MetaCore and co-expression network respectively. Distinct network modules and nodes were identified based on the three sub-networks. For each sub-network and module, GO and KEGG pathway enrichment were achieved employing DAVID. Finally, miRNAs potentially involved in AD-like phenotype in SAMP8 mice were analyzed using experimental validated miRNA target data.

**Figure 2. The distinct genetic sub-network of hippocampus and cerebral cortex in SAMP8 mice based on PPI network.** Red nodes represent differential expressed genes in SAMP8 mice, green nodes are intermediate genes, and genes marked with blue circle are AD-related. The size of the node corresponds to the number of its direct neighbors. The nodes are linked if they have interaction in PPI network.

**Figure 3. The distinct genetic sub-network of hippocampus and cerebral cortex in SAMP8 mice based on** **MetaCore network.** Red nodes represent differential expressed genes in SAMP8 mice, green nodes are intermediate genes, and genes marked with blue board are AD-related. The size of the node corresponds to the number of its direct neighbors. The nodes are linked if they have interaction in MetaCore. The red line with black arrow indicates activation. The blue line with black small line segments indicates inhibition. The blue line indicates there is undefined interaction between nodes.

**Figure 4. The distinct genetic sub-network of hippocampus and cerebral cortex in SAMP8 mice based on co-expression network.** Red nodes represent differential expressed genes in SAMP8 mice, green nodes are intermediate genes, and genes marked with blue board are AD-related. The size of the node corresponds to the number of its direct neighbors. The nodes are linked if they are significantly co-expressed.

**Figure 5. The remarkable biological processes and pathways in the brain of SAMP8 mice.** GO functional annotation (left) and KEGG pathway enrichment (right) of PPI, co-expression and MetaCore sub-networks.

**Figure 6. The significant modules and nodes of genetic sub-network in the brain of SAMP8 mice.** Red nodes are differential expressed genes in SAMP8 mice, and green nodes are intermediate genes. The genes are linked with red line if they have interaction in MetaCore, green line if they have interaction in PPI, and blue line if they are significant co-expressed. The red line with black arrow indicates activation. The red line with black small line segments indicates inhibition.

**Figure 7. The function of significant modules and nodes** **of genetic sub-network in the brain of SAMP8 mice.** GO functional annotation (left) and KEGG pathway enrichment (right) of 10 modules. Red rectangle nodes are modules, and blue round nodes are GO terms and KEGG pathways respectively. The size of the node corresponds to its direct neighbors.

**Figure 8. MicroRNAs targeting on nodes of genetic sub-network in the brain of SAMP8 mice.** In this miRNA-gene bipartite graph network, round nodes represent genes in the PPI, co-expression, and MetaCore sub-network in the brain of SAMP8, blue round nodes are differential expressed genes in SAMP8 mice, and green round nodes are intermediate genes. Red triangle nodes represent miRNAs targeting on these genes. The links represent validated target interactions.
