# Supplementary material for: Nodes and biological processes identified on the basis of network analysis in the brain of the senescence accelerated mice as an Alzheimer's disease animal model
Source: Front Aging Neurosci. 2013 Oct 29;5:65. doi: 10.3389/fnagi.2013.00065 (PMC3810591; doi:10.3389/fnagi.2013.00065)
Supplement: Supplementary file 1 [file DataSheet1.ZIP › Supplementary information and Figure legends/Supporting Information.doc]

**Supporting Information**

| The biological background network | Mapping seed genes to the background network | The sub-network in the brain of SAMP8 mice |
| --- | --- | --- |
|  | | |
| **Supplement figure 1. The schematic diagram of network inference procedure.** Gray node is gene in database (HPRD, COXPRESdb, or Meta-Core). Red node is seed gene (differential expression genes in the brain of SAMP8 mice). Green node is intermediate gene (genes in HPRD, COXPRESdb, or Meta-Core). | | |

A

B

**Supplement figure 2. Sub-network in SAMP8 mice based on PPI network.** (A) Sub-network of hippocampusin SAMP8 mice. (B) Sub-network of cerebral cortex in SAMP8 mice. Red nodes represent differential expressed genes in SAMP8 mice, green nodes are intermediate genes, and genes marked with blue circle are AD-related. The size of the node corresponds to the number of its direct neighbors. The nodes are linked if they have interaction in PPI network.

A

B

**Supplement figure 3. Sub-network in SAMP8 mice based on MetaCore network.** (A) Sub-network of hippocampus in SAMP8 mice. (B) Sub-network of cerebral cortex in SAMP8 mice. Red nodes represent differential expressed genes in SAMP8 mice, green nodes are intermediate genes, and genes marked with blue circle are AD-related. The size of the node corresponds to the number of its direct neighbors. The nodes are linked if they have interaction in MetaCorenetwork. The red line with black arrow indicates activation. The blue line with black small line segments indicates inhibition. The blue line indicates there isundefined interaction between nodes.

A

B

**Supplement figure 4. Sub-network in SAMP8 mice based on co-expression network.** (A) Sub-network of hippocampus in SAMP8 mice. (B) Sub-network of cerebral cortex in SAMP8 mice. Red nodes represent differential expressed genes in SAMP8 mice, green nodes are intermediate genes, and genes are marked with blue board if they are AD-related. The size of the node corresponds to the number of its direct neighbors. The nodes are linked if they are significantly co-expressed.
